# Carcinogens in Rat Milk

**DOI:** 10.1038/bjc.1974.187

**Published:** 1974-09

**Authors:** R. Schoental, T. A. Gough, K. S. Webb

## Abstract

Mothers of 5-day old rats were given diethylnitrosamine (DEN) (130 mg/kg body weight) by stomach tube. The milk removed from the stomachs of the suckling young contained 5, 16 and 36 parts/10^6^ of DEN at 2, 4 and 6 hours respectively after they started suckling the treated mothers. After 49 hours, DEN was no more detectable in the milk.


*Br. J. Cancer* (1974) **30**, 238

### CARCINOGENS IN RAT MILK

### TRANSFER OF INGESTED DIETHYLNITROSAMINE INTO MILK BY LACTATING RATS

R. SCHOENTAL*, T. A. GOUGH† AND K. S. WEBB†

*From the *Department of Pathology, Royal Veterinary College, London NW1 0TU and the †Laboratory of the Government Chemist, Department of Trade and Industry, Cornwall House, Stamford Street, London SE1 9NQ*

Received 1 April 1974. Accepted 16 May 1974

**Summary.—Mothers of 5-day old rats were given diethylnitrosamine (DEN) (130 mg/kg body weight) by stomach tube. The milk removed from the stomachs of the suckling young contained 5, 16 and 36 parts/$10^6$ of DEN at 2, 4 and 6 hours respectively after they started suckling the treated mothers. After 49 hours, DEN was no more detectable in the milk.**

ALKYLNITROSAMINES are among the most versatile and potent carcinogens, able to induce tumours in various organs of several animal species (Magee and Barnes, 1967; Druckrey *et al.*, 1967).

No direct evidence is available to indicate whether nitrosamines could be carcinogenic in man, but in workers exposed industrially to dimethylnitrosamine (DMN), acute liver damage and cirrhosis have been observed (Freund, 1937); there is little doubt that under certain conditions nitroso compounds could represent health hazards (Magee, 1971).

Search for nitrosamines in foodstuffs is fraught with difficulties, due to the possibility of false positives, artefacts or losses in the course of the analytical procedures. Volatile nitrosamines have been detected in various foodstuffs (Ender *et al.*, 1964; Hedler and Marquardt, 1968; Fazio *et al.*, 1971; Fong and Walsh, 1971; Crosby *et al.*, 1972 and others).

In view of the great susceptibility of the very young to many carcinogens, including the alkylnitrosamines (for references see Magee and Barnes, 1967; Druckrey *et al.*, 1967; Schoental, 1974*a*), particular attention is required to ensure that milk, the main foodstuff of the young, does not contain carcinogenic nitroso compounds.

Cow's milk and its products were tested by several groups of workers but gave variable results. Traces of diethylnitrosamine (DEN) have been detected in pasteurized milk and in Tilsit cheese (Hedler and Marquardt, 1968) and of DMN in several varieties of cheese (Crosby *et al.*, 1972). On the other hand, when extracts from fat-free heat dried milk were examined (after oxidation to the respective nitramines) by electron capture gas chromatography on Rheoplex 400 columns, a peak coinciding with the oxidation products of DMN has been observed. However, when re-chromatographed on OV-1 column, the material from milk differed from the reference sample of the oxidation product of DMN (Reineccius and Coulter, 1972).

Another approach to the subject is to determine whether and under which experimental conditions volatile alkylnitrosamines, administered to lactating animals, would appear in the milk, in what concentration and whether this could account for the induction of tumours in the offspring. The possibility that carcinogenic metabolites of the parent compound

(Blattmann and Preussman, 1973) may be excreted in the milk has also to be considered.

In our exploratory experiments, when nursing rats were given a few doses of DEN (130 mg/kg body weight) various tumours, including aesthesioneuroepitheliomata developed in the suckling young (Schoental and Appleby, 1973; Schoental, 1974*b*).

In the present communication we report the finding of free DEN in the milk removed from the stomach of the suckling young at various times within a few hours after administration of DEN to their mothers.

### EXPERIMENTS AND RESULTS

Three white, mother rats with their litters (8–11 each) random bred from the Wistar–Porton strain were received from the M.R.C. Laboratory Animals Centre, Carshalton 5 days after parturition. Two of the mothers were given DEN (40 mg each in 0·5 ml of 10% aqueous ethanol) by stomach tube and were kept for about 30 min away from their young in order to avoid direct transfer of DEN to the young on being licked by the mothers.

At 2, 4, 6 and 49 h after the mother rats had been returned to their offspring, 5 of the young rats were killed by decapitation, the stomachs were dissected out and their content (consisting of clotted milk) removed, pooled and kept in glass bottles in deep freeze (at −20°C) until it could be analysed.

Five of the offspring of the third mother rat, kept as controls, were killed when 7 days old, corresponding to the age of the experimental ones killed at 49 h after the beginning of the experiment. The milk removed from their stomachs was tested as negative control, in a similar way to that of the experimental sucklings.

The milk samples were extracted with 1 ml of dichloromethane (DCM) and 5 $\mu$l of this DCM extract was injected without further clean up on to a Philips model R gas chromatograph (GC) linked to an AEI MS902 mass spectrometer. The GC was equipped with a 2·4 m × 2 mm ID GC column containing 15% carbowax 20M in series with a 5·4 m × 2 mm ID column containing 5% carbowax 20M, the stationary phase being supported on 80–100 mesh AW Chromosorb W on both columns. The GC oven temperature was 145°C. In order to separate the helium carrier gas from the GC eluant before entering into the mass spectrometer, a membrane separator (Gough and Webb, 1972) was used and a solvent venting device (Gough and Webb, 1973) was incorporated in the GC to prevent pressure surges in the mass spectrometer.

The presence of DEN in the extracts was established by parent ion monitoring (Gough and Webb, 1972) with a mass spectrometer resolution of 7000, using perfluorotributylamine as a reference material. The results given below are precise to within 10% based on the final extract.

| Milk (h) | DEN (mg/l) |
|---|---|
| 2 | 5 |
| 4 | 15 |
| 6 | 36 |
| 49 | None |
| Control | None |

The limit of detection for DEN was 1 mg/l.

There was no evidence for the presence of free volatile metabolic derivatives of DEN.

### DISCUSSION

It is of interest that significant concentrations of unchanged free DEN were found in the milk of suckling rats within a few hours after their mothers were given DEN (130 mg/kg) by stomach tube. Exact measurements of the amounts of DEN ingested with milk by the young are difficult to obtain. The young were left with their mothers until shortly before administering the DEN, hence the stomachs of the young would contain some “ clean ” milk which would suppress the DEN concentration during the first few hours after administration. Diffusion of DEN from the stomach and digestion would also affect the accuracy of the data. However, on the basis that a suckling rat of 6–10 g body weight would receive about 1 ml of milk within the time that DEN was present in the mother's milk, a dose of about 20 μg of DEN would be received by the offspring. In a parallel experiment in which the mothers were given several doses of DEN (each 130 mg/kg), the suckling rats were allowed to survive. They grew and developed in apparent good health until tumours developed later in life (Schoental, 1974*b*). On the basis of the present observations, the formation of the tumours in the offspring can be attributed, at least in part, to the repeated ingestion of unchanged DEN during lactation.

We thank Dr J. M. Barnes for the gift of diethylnitrosamine which was used in these experiments. One of us (R.S.) is indebted to Professor E. Cotchin for hospitality in his Department.

### REFERENCES

Blattmann, L. & Preussmann, R. (1973) Structure of Rat Urinary Metabolites of Carcinogenic Dialkylnitrosamines. *Z. Krebsforsch.*, **79**, 3.

Crosby, N. T., Foreman, J. K., Palframan, J. F. & Sawyer, R. (1972) Estimation of Steam-volatile N-nitrosamines in Foods at the 1 μg/kg Level. *Nature, Lond.*, **238**, 342.

Druckrey, H., Preussmann, R., Ivankovic, S. & Schmähl, D. (1967) Organotrope carcinogene Wirkung bei 65 verschiedenen N-Nitroso-Verbindungen an BD Ratten. *Z. Krebsforsch.*, **69**, 103.

Ender, F., Havre, G., Helgebostad, A., Koppang, N., Madsen, R. & Ceh, L. (1964) Isolation and Identification of a Hepatotoxic Factor in Herring Meal Produced from Sodium Nitrite Preserved Herring. *Naturwissenschaften*, **51**, 637.

Fazio, T., Damico, J. N., Howard, J. W., White, R. H. & Watts, J. O. (1971) Gas Chromatographic Determination and Mass Spectrometric Confirmation of *N*-nitrosodimethylamine in Smoke-processed Marine Fish. *J. agric. Fd Chem.*, **19**, 250.

Fong, Y. Y. & Walsh, E. O'F. (1971) Carcinogenic Nitrosamines in Cantonese Salt-dried Fish. *Lancet*, ii, 1032.

Freund, H. A. (1937) Clinical Manifestation and Studies in Parenchymatous Hepatitis. *Ann. intern. Med.*, **10**, 1144.

Gough, T. A. & Webb, K. S. (1972) The Use of Molecular Separator in Determination of Trace Constituents by Combined Gas Chromatography and Mass Spectrometry. *J. Chromat.*, **64**, 201.

Gough, T. A. & Webb, K. S. (1973) A Method for the Detection of Traces of Nitrosamines using Combined Gas Chromatography and Mass Spectrometry. *J. Chromat.*, **79**, 57.

Hedler, L. & Marquardt, P. (1968) Occurrence of Diethylnitrosamine in Some Samples of Food. *Fd & Cosmet. Toxicol.*, **6**, 341.

Magee, P. N. (1971) Toxicity of Nitrosamines: their Possible Human Health Hazards. *Fd & Cosmet. Toxicol.*, **9**, 207.

Magee, P. N. & Barnes, J. M. (1967) Carcinogenic Nitroso Compounds. *Adv. Cancer Res.*, **10**, 163.

Reineccius, G. A. & Coulter, S. T. (1972) Examination of Nonfat Dry Milk for the Presence of Nitrosamines. *J. Dairy Sci.*, **55**, 1574.

Schoental, R. (1974*a*) Carcinogenicity as Related to Age. *Ann. Rev. Pharmacol.*, **14**, 185.

Schoental, R. (1974*b*) Tumours in Rats Suckled by Mothers Treated with Carcinogens during Lactation. *Proc. Second Int. Symp. Cancer Detection and Prevention*, Bologna. (1973) In the press.

Schoental, R. & Appleby, E. C. (1973) The Development of Tumours in a Female Rat and her Offspring, following Administration of Diethylnitrosamine to the Mother during Nursing. *Br. J. Cancer*, **28**, 84.


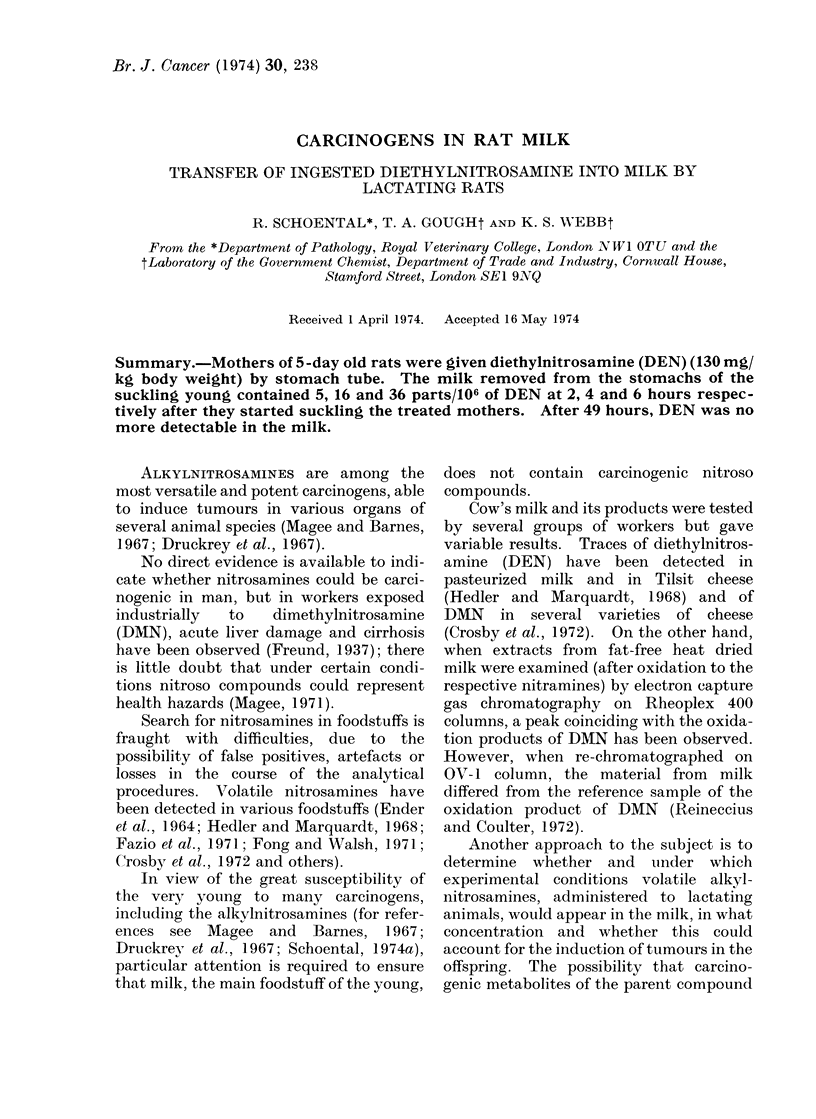

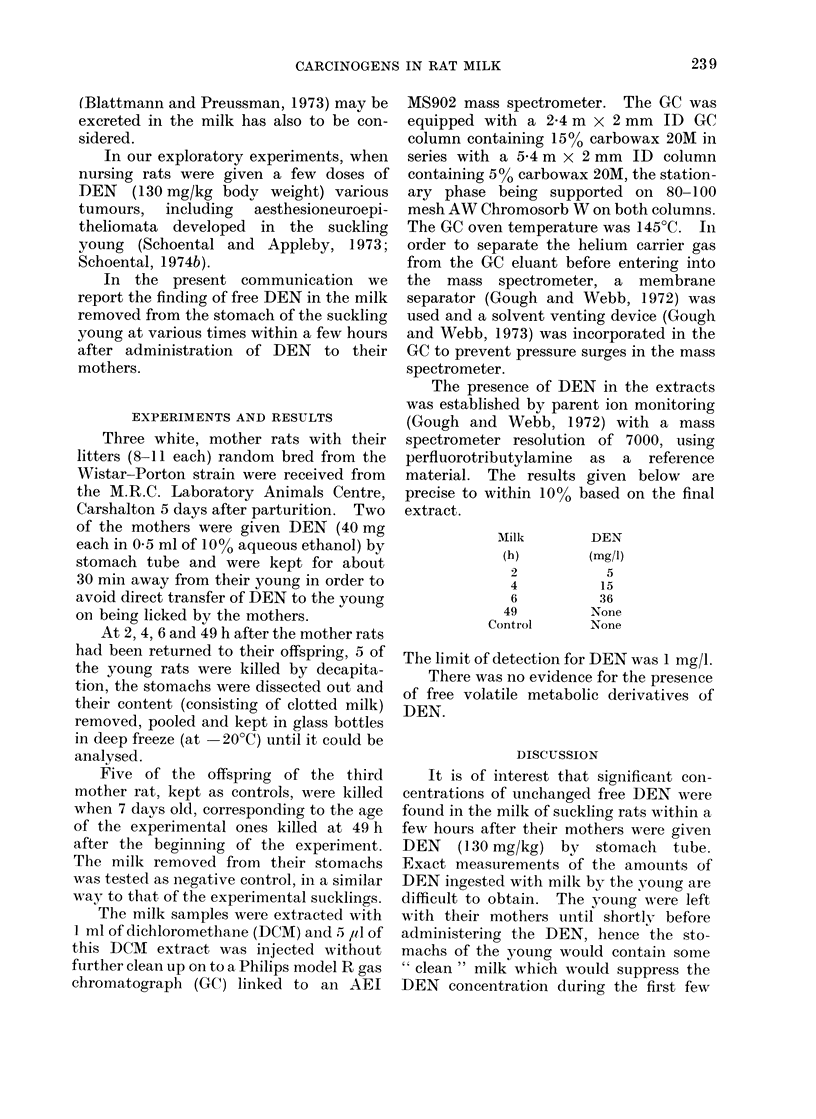

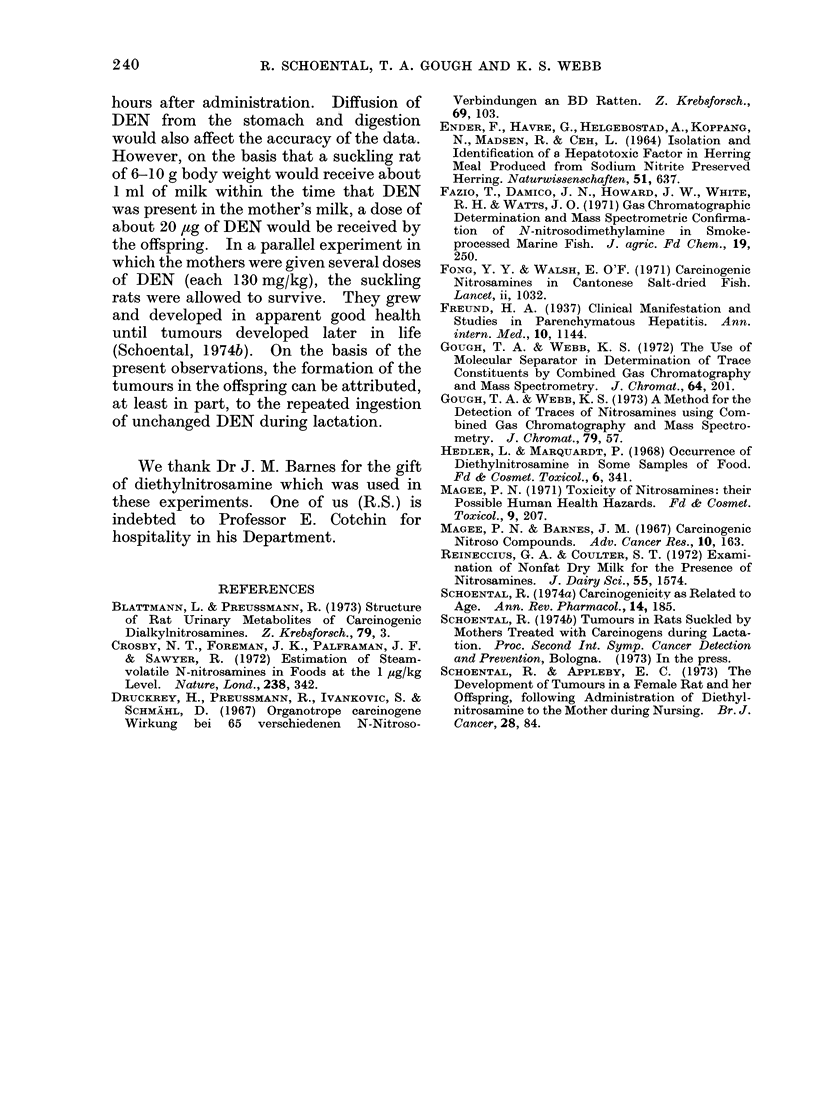


## References

[OCR_00252] Blattmann L., Preussmann R. (1973). Struktur von Metaboliten carcinogener Dialkylnitrosamine im Rattenurin. Z Krebsforsch Klin Onkol Cancer Res Clin Oncol.

[OCR_00257] Crosby N. T., Foreman J. K., Palframan J. F., Sawyer R. (1972). EWstimation of steam-volatile N-nitrosamines in foods at the 1 micro g-kg level.. Nature.

[OCR_00263] Druckrey H., Preussmann R., Ivankovic S., Schmähl D. (1967). Organotrope carcinogene Wirkungen bei 65 verschiedenen N-Nitroso-Verbindungen an BD-Ratten.. Z Krebsforsch.

[OCR_00278] Fazio T., Damico J. N., Howard J. W., White R. H., Watts J. O. (1971). Gas chromatographic determination and mass spectrometric confirmation of N-nitrosodimethylamine in smoke-processed marine fish.. J Agric Food Chem.

[OCR_00286] Fong Y. Y., Walsh E. O. (1971). Carcinogenic nitrosamines in Cantonese salt-dried fish.. Lancet.

[OCR_00302] Gough T. A., Webb K. S. (1973). A method for the detection of traces of nitrosamines using combined gas chromatography and mass spectrometry.. J Chromatogr.

[OCR_00308] Hedler L., Marquardt P. (1968). Occurrence of diethylnitrosamine in some samples of food.. Food Cosmet Toxicol.

[OCR_00318] Magee P. N., Barnes J. M. (1967). Carcinogenic nitroso compounds.. Adv Cancer Res.

[OCR_00313] Magee P. N. (1971). Toxicity of nitrosamines: their possible human health hazards.. Food Cosmet Toxicol.

[OCR_00322] Reineccius G. A., Coulter S. T. (1972). Examination of nonfat dry milk for the presence of nitrosamines.. J Dairy Sci.

[OCR_00337] Schoental R., Appleby E. C. (1973). The development of tumours in a female rat and her offspring, following administration of diethylnitrosamine to the mother during nursing.. Br J Cancer.

